# Revitalizing elixir with orange peel amplification of alginate fish oil beads for enhanced anti-aging efficacy

**DOI:** 10.1038/s41598-024-71042-w

**Published:** 2024-09-02

**Authors:** Archna Dhasmana, Subham Preetam, Sumira Malik, Vikash Singh Jadon, Nupur Joshi, Geeta Bhandari, Sanjay Gupta, Richa Mishra, Sarvesh Rustagi, Shailesh Kumar Samal

**Affiliations:** 1 Himalayan School of Biosciences, Swami Rama Himalayan University, Swami Ram Nagar, Jollygrant, Dehradun, Uttarakhand India 248140; 2 Department of Robotics and Mechatronics Engineering, Daegu Gyeongbuk Institute of Science and Technology (DGIST), Dalseong-gun, Daegu, 42988 South Korea; 3 Amity Institute of Biotechnology, Amity University Jharkhand, Ranchi, 834002 India; 4 Department of Computer Engineering, Parul Institute of Engineering and Technology ( PIET), Parul University, Ta. Waghodia, Vadodara, Gujarat 391760 India; 5 School of Applied and Life Sciences, Uttaranchal University, 22 Dehradun, Uttarakhand India; 6 Unit of Immunology and Chronic Disease, Institute of Environmental Medicine, Karolinska Institutet, 171 77 Stockholm, Sweden

**Keywords:** Hydrogel beads, Anti-aging, Antioxidants, Anti-inflammatory, Drug delivery, Biotechnology, Drug discovery, Neuroscience, Health care

## Abstract

The research introduces a novel method for creating drug-loaded hydrogel beads that target anti-aging, anti-oxidative, and anti-inflammatory effects, addressing the interconnected processes underlying various pathological conditions. The study focuses on the development of hydrogel beads containing anti-aging compounds, antioxidants, and anti-inflammatory drugs to effectively mitigate various processes. The synthesis, characterization and in vitro evaluations, and potential applications of these multifunctional hydrogel beads are discussed. A polymeric alginate-orange peel extract (1:1) hydrogel was synthesized for encapsulating fish oil. Beads prepared with variable fish oil concentrations (0.1, 0.3, and 0.5 ml) were characterized, showing no significant decrease in size i.e., 0.5 mm and a reduction in pore size from 23 to 12 µm. Encapsulation efficiency reached up to 98% within 2 min, with controlled release achieved upto 45 to 120 min with increasing oil concentration, indicating potential for sustained delivery. Fourier-transform infrared spectroscopy confirmed successful encapsulation by revealing peak shifting, interaction between constituents. In vitro degradation studies showed the hydrogel's biodegradability improved from 30 to 120 min, alongside anti-inflammatory, anti-oxidative, anti-collagenase and anti-elastase activities, cell proliferation rate enhanced after entrapping fish oil. In conclusion, the synthesized hydrogel beads are a promising drug delivery vehicle because they provide stable and effective oil encapsulation with controlled release for notable anti-aging and regenerative potential. Targeted delivery for inflammatory and oxidative stress-related illnesses is one set of potential uses. Further research may optimize this system for broader applications in drug delivery and tissue engineering.

## Introduction

Ageing is a complex biological process driven by inflammation and oxidative stress that is characterised by a decline in physiological activities and an increased vulnerability to age-related illnesses. Cellular damage is caused by oxidative stress, that results due to the imbalance between the creation of reactive oxygen species and the native antioxidative defences. Chronic inflammation exacerbates this and speeds up tissue malfunction. Polypharmacy is a common part of conventional therapy, which increases the risk of side effects and limited compliance. As a result, there is a growing emphasis on multifunctional medicines that target multiple ageing pathways at once to manage age-related disorders more successfully^1^.

Initially acting as a defensive reaction, inflammation can develop into a chronic condition called inflammation, which accelerates ageing and exacerbates several age-related illnesses, including cancer, neurological diseases, cardiovascular problems, and ageing of the skin^2^. Visible indicators of ageing such as wrinkles and sagging are caused by the chronic inflammation-induced degradation of collagen and elastin fibres in the skin^3^. Thus, anti-inflammatory and anti-inflammatory therapies, as well as lifestyle changes, have the potential to reduce age-related diseases and encourage good ageing by addressing inflammation and oxidative stress^4,5^. These approaches may provide ways to postpone the onset of age-related disorders and increase longevity by reducing the damaging effects of environmental exposure and chronic inflammation on aging-related processes.

Inflammation is categorized as either acute or chronic based on its duration of persistence^6^. While both types play beneficial roles in the body's healing, they can also bring temporary discomfort like pain, soreness, and swelling. Chronic inflammation poses a more severe challenge, potentially leading to various diseases such as diabetes, cancer, and arthritis due to prolonged tissue damage and internal bleeding^7,8^. Thus, there is a pressing need for anti-inflammatory strategies to address these issues and support healing processes.

Although NSAIDs and other chemical medications are commonly used to treat inflammation, their effectiveness is limited by gastrointestinal adverse effects^9^. As a result, people are becoming more interested in natural substitutes that are thought to be safer, such as nutritional and herbal supplements. Even though they come from different places, natural medicines and NSAIDs often work by blocking the nuclear factor pathway and COX inflammatory activity^10,11^. This search is part of a larger trend towards therapies with similar efficacy but fewer side effects.

Research shows natural anti-inflammatory drugs inhibit NF-kB activation and other inflammatory mediators. Polyphenolic compounds present in citrus fruit peels, turmeric, white willow bark, green tea, and omega-3 fatty acids from fish oil offer significant benefits without side effects^12^. Encapsulation enhances their therapeutic effects, ensuring stability and controlled release, making it a valuable drug delivery system.

Fish oil, enriched with omega-3 polyunsaturated acids such as eicosapentaenoic acid (EPA) and docosahexaenoic acid (DHA), has shown considerable anti-inflammatory activity, making it beneficial for treating inflammation-related ailments^13^. Methods such as enzymatic extraction and green extraction techniques using CO_2_ for supercritical fluid extraction (SFC-CO_2_), microwave-assisted extraction (MAE), and ultrasound-assisted extraction (UAE) have been favored over traditional methods due to their optimization of yield and productivity, shorter extraction times, and lower operating temperatures^14^. However, the implementation of fish oil has been limited by its foul odor and susceptibility to rancidity when exposed to air and moisture.

Hydrogels have become highly flexible drug delivery vehicles because of its special qualities such as biocompatibility, their high-water content, mechanical properties that may be adjusted, and capacity to encapsulate a variety of bioactive agents^15^. Drug delivery systems based on hydrogels provide regulated release of medicinal substances, increasing effectiveness while reducing negative effects^16^. Through the integration of anti-aging agents, antioxidants, and anti-inflammatory medications into hydrogel matrix, multifunctional formulations that target several facets of the ageing process can be created.

Polymeric matrices play a pivotal role in encapsulating essential oils, offering protection, controlled release, and enhanced bioavailability^17,18^. Essential oils, sensitive to environmental factors, require safeguarding to maintain efficacy. Polymeric barriers shield oils from degradation, extending shelf life. Controlled release is facilitated by tailored polymer properties, ensuring sustained supply and optimized therapeutic effects. Enhanced bioavailability is achieved through improved solubility and absorption. Polymeric matrices have flexible and compatible polymers that accommodate various oils and delivery systems and also mask unpleasant odours and flavours, expanding application possibilities. In summary, polymeric matrices serve as effective vehicles for essential oil encapsulation, benefiting pharmaceutical, cosmetic, and food industries.

Gel beads offer a versatile and efficient platform for the encapsulation of oils, providing several advantages including controlled release, enhanced stability, and ease of handling. Gel beads are typically composed of hydrophilic polymers such as sodium alginate or gelatin, which form a crosslinked network upon exposure to divalent cations like calcium ions. Overall, gel beads offer a convenient and effective means of encapsulating oils, providing controlled release, enhanced stability, and versatility for various applications in the pharmaceutical, cosmetic, and food industries. Their unique properties make them ideal candidates for encapsulating oils and delivering them in a controlled and efficient manner.

## Material and methods

### Hydrogel bead preparation

Orange (*Citrus reticulata*) peel extraction (OPE) was done with a straightforward washing process under running water, followed by drying. Subsequently, 1000 g of the separated peel was subjected to ethanol extraction process of 70%^19^. Fish oil was obtained by extracting the oil content from cod liver oil capsules. Two grams of sodium alginate were then measured and dissolved in 100 mL of distilled water with the assistance of a magnetic stirrer.

**Fig. 1:**
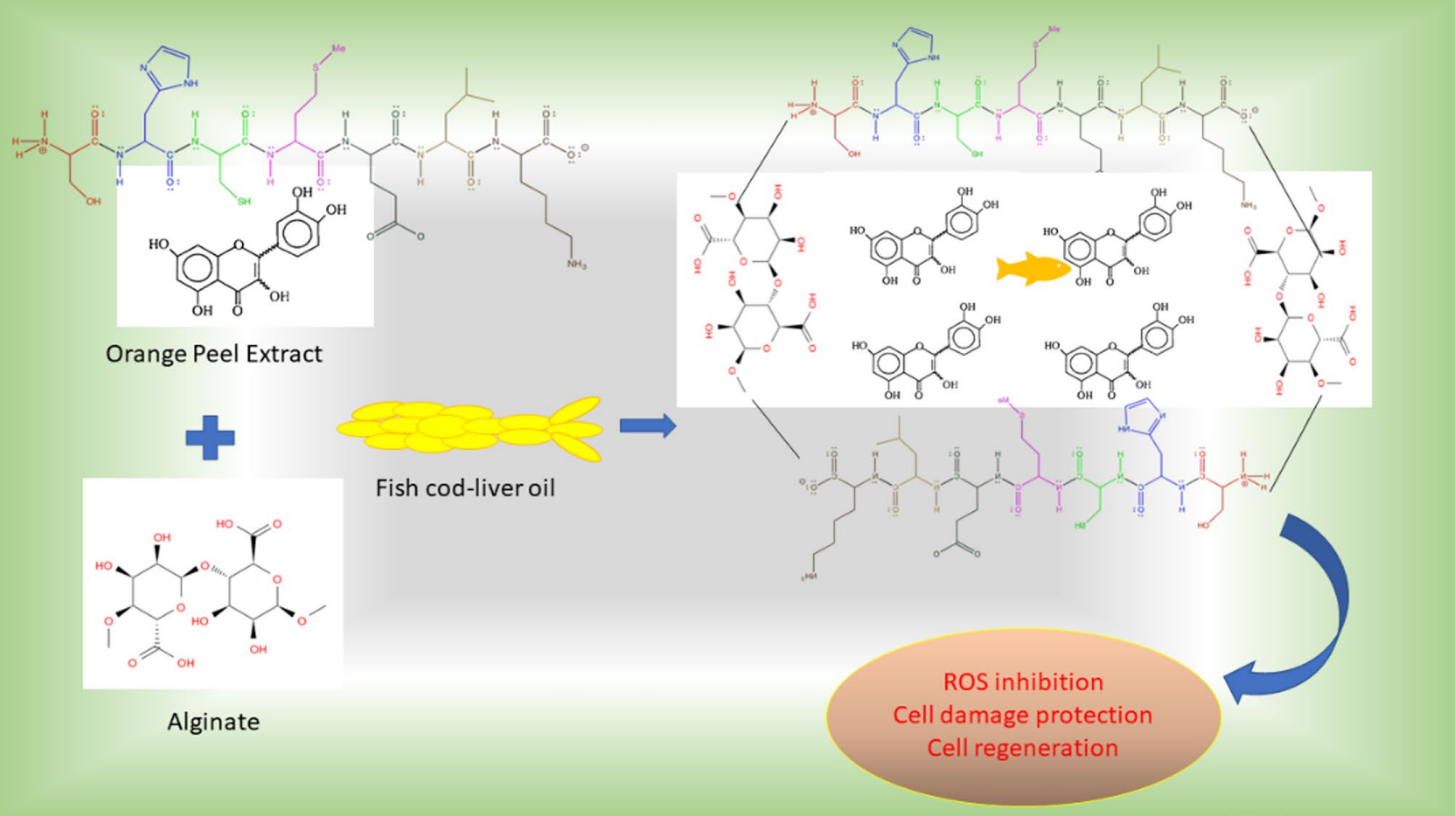
Schematic representation of the oil encapsulated alginate-orange peel extract (OPE) beads for the enhanced antiaging effect and cell regeneration potential.

The schematic representation of the drug (fish oil) encapsulated Alginate hydrogel bead preparation methodology is illustrated in Fig. 1 and Table 1. Initially, 2 g of orange peel extract was added to the alginate solution and thoroughly mixed to ensure even dispersion. The resulting mixture was then transferred to a syringe and added drop by drop into a solution containing different concentrations of fish oil and 1% CaCl_2_. After 2 min, samples were retrieved from the solution, resulting in the formation of hydrogel beads. The beads formed solely in the CaCl_2_ solution were designated as the control, while those formed in the fish oil-containing CaCl_2_ solution were considered as drug-loaded beads. Relative hydrogel beads were prepared similarly for the corresponding discrete samples. Each sample's composition was tailored to achieve specific properties and functionalities in the resulting hydrogel beads, aiming to optimize their performance for anti-inflammatory and tissue remodeling applications.

**Table 1:** Composition of the polymeric hydrogel matrix prepared for the entrapment of oil.

Composition (per 100 ml solution)	Control	Sample 1	Sample 2	Sample 3
Alginate (gm)	2%			
OPE (gm)	2%			
Fish oil (ml)	–	0.1	0.3	0.5

The synthesis of hydrogel beads involved a crosslinking polymerization technique. Initially, hydrogel precursors, which could be natural or synthetic polymers (e.g., alginate, chitosan, polyethylene glycol), were dissolved in an aqueous solution containing the desired drugs or bioactive compounds. Crosslinking agents such as calcium ions, glutaraldehyde, or photo-initiators were then added to trigger gelation and form spherical beads.

### Characterization of the fabricated drug loaded scaffold

The characterization process of hydrogel beads involves assessing various properties to gain insights into their structure–property relationships and optimize their formulation for specific applications like wound healing and tissue remodeling. Key aspects typically evaluated during this process include understanding the structure, composition, and performance of the beads.

#### Size and morphology

The morphological structure analysis of the citrus extract and fish oil encapsulated within alginate hydrogel beads was conducted through straightforward visualization i.e., macro and microscopic observation. The size of the beads was measured using a vernier caliper to determine their diameter in millimeters (mm). Measurement of bead diameter using optical microscopy or image analysis software, and ultrastructure of bead morphology, including shape and surface features was done using field emission scanning electron microscopy (FESEM).

#### Functional group analysis

FTIR spectra analysis was conducted to examine the functional groups and chemical interactions among the components of the beads^20^. This qualitative analysis involved preparing compressed pellets of the sample with potassium bromide (at a ratio of 1:900 mg) and recording absorbance in the range of 4000 to 400 cm^−1^ using an FTIR spectrophotometer (Thermo Nicolet, USA).

### Oil encapsulation efficiency

Determination of the amount of active ingredient (fish oil) encapsulated within the beads was done by the standard protocol given in the literature^21^. Calculation of encapsulation efficiency as the percentage of drug content encapsulated relative to the total amount used in the formulation. The encapsulation efficiency was determined by the following formula.

$$Encapsultion\; efficiency\; (\%)=(AQ/TQ)\times 100$$

where AQ is the actual drug content of beads and TQ is the theoretical quantity of drug present in beads.

### In vitro stability and oil release profile

The prepared gel beads were immersed in a phosphate-buffered saline (PBS) solution with a pH maintained at 7.2 in a shaker incubator to provide mechanical agitation (50 RPM), thereby the degradation rate enhance and mimic the biological system by increasing the surface area exposed to the degrading medium^22,23^. To assess biodegradability, beads were withdrawn at intervals of 10 min, weighed, and the percentage weight loss was calculated, further release profile checked to confirm the degradation of polymeric matrix. The percentage weight loss was determined using the following equation:

$$Weight\; loss\, (\%)=\left(wi-wf|wi\right)\times 100$$

The pH value of the resultant PBS solution was also measured using pH meter at different time intervals. The swelling rate also measured after regular time interval to measure the rate of absorption of the beads.

However, the characteristics of active fish oil as drug release from the hydrogel beads were assessed using a total immersion process^24^. UV–Vis spectra were recorded utilizing a UV–Vis spectrophotometer to prepare calibration curves and monitor the drug release over time.

### In vitro profiling

The efficacy of the fish oil-loaded hydrogel beads was evaluated in vitro using cell culture models to assess their anti-aging, antioxidative, and anti-inflammatory effects. Cell viability assays, ROS assays, measurement of inflammatory markers, and FESEM analysis were performed to evaluate the therapeutic efficacy and biocompatibility of the hydrogel formulations^24,25^.

### Anti-inflammatory activity

The inflammatory inhibition activity of the prepared samples was estimated performing the 5-LOX (Lipooxygenase) inhibition assay by following the standard protocol. Briefly, make 3 mg beads containing the different concentration fish oil with 30 μL of linoleic acid and potassium phosphate buffer (0.1 M, pH 6.3) containing 5-LOX (25 U). Here, the linoleic acid acts as substrate for 5-LOX enzyme to convert into a conjugated diene by 5-LOX, which results in a continuous increase in absorbance at 234 nm. All the prepared mixtures kept at 25℃ for 10 min, and the absorbance was measured at 234 nm. The inhibition activity was calculated as Anti-Inflammatory activity (Infl (%)) by the following the equation as mentioned below:

$$Infl (\%)=\left\{(Ab-As)/Ab\right\}\times 100$$

where Ab is the absorbance of blank and As the absorbance of the sample. Here, control sample was taken sodium phosphate buffer (blank) and DMSO into the quartz cuvette.

### Anti-oxidative study

To measure anti-oxidative effect of the prepared samples 2, 2-diphenyl-1-picrylhydrazyl (DPPH) assay methods was used, thereby we can estimate the efficiency to prevent the free-radicals and reactive oxygen species generation. In brief the samples at variable concentration (1 μg to 10 μg /mL) were treated with the prepared reagents followed by 30 min incubation in the dark condition and checked the absorbance at 517 nm. Afterward, the absorbance of the sample measured at 517 nm and calculates the anti-oxidative activity (AA) by the formula mentioned below:

$$\it AA(\%)=100-(\text{Abs sample}-\text{Abs control}/\text{Abs control})\times 100$$

In DPPH assays, distilled water was used as a blank, quercetin (125 µg/mL) as standard, and the findings were reported as percent inhibition, respectively (Fig. 6B).

### Anti-aging activity

The fundamental constituents of human skin health, collagen and elastase provide skin its structural integrity, elasticity, firmness, and flexibility. The freshly produced bead samples were tested for anti-collagenase and anti-elastase activity in order to verify the anti-aging effect reported in the literature^26^. As we age, wrinkles are caused by the breakdown of collagen in the skin by enzymes called collagenases. Briefly, the collagenase enzyme (cell culture grade) was obtained from Clostridium histolyticum, while the positive control (EGCG) and negative control (aborbic acid) were employed. Following a 20 min collagenase enzyme incubation period, N-[3-(2-furyl)acryloyl]-Leu Gly-Pro-Ala (FALGPA) was added as a substrate to fresh bead samples. To calculate the anti-aging impact, the absorbance at 340 nm was measured after incubation using microplate reader.

$$Collagenase\; inhibition\, (\%)= \left\{\left(A-B\right)-\left(C-D\right)\div 100\right\}\times 100$$

Here, A is the UV absorbance of collagenase solution combined with the FALGPA solution, B is the UV absorbance of the solvents, C is the UV absorbance of the bead samples (control and oil entrapped beads) combined with the collagenase and FALGPA solution, and D is the UV absorbance of the bead samples solution. All experiments were done in triplicate.

Subsequently, an elastase inhibition experiment was used to determine anti-elastase activity. In brief the elastase enzyme was applied to fresh bead samples along with N-succinyl-triallyl-p nitroanilide (SANA) as a substrate. In this case, dimethyl sulfoxide (DMSO) was utilized as the negative control and EGCG as the positive control. In advance of utilising a microplate reader to measure the absorbance at 734 nm, all enzyme-treated samples had a pre-incubation period at room temperature and were treated with SANA.

$$Elastase\; inhibition (\%)= \left\{\left(A-B\right)-\left(C-D\right)\div 100\right\}\times 100$$

Here A is the UV absorbance of collagenase solution combined with the SANA solution, B is the UV absorbance of the solvents, C is the UV absorbance of the bead samples (control and oil entrapped beads) combined with the collagenase and FALGPA solution, and D is the UV absorbance of the bead samples solution. All experiments were done in triplicate.

### Cytocompatibility

The biocompatibility of prepared samples and their synergistic effect on cell growth has been tested on L929 fibroblast cell by following the protocol^24^. In brief the prepared control and gel beads socked in cell culture growth medium and incubated for 2 h in CO_2_ incubator. Following the incubation, the nutrient media were removed, and 10 µl of cell suspension (1 × 10^3^ cells/well) was added onto the moist matrix. Subsequently, 990 µl of freshly prepared DMEM medium was added to each well, and the tissue culture plate was then placed in the CO_2_ incubator at 37 °C. The MTT assay was conducted at regular intervals by measuring absorbance at a wavelength of 490 nm using a Microplate Reader (TECAN, India) to assess cell viability and proliferation.

Furthermore, FESEM analysis of the cell seeded matrix was performed to confirm cell adherence and spreading over the scaffold, following a previous protocol. After 20 min incubation cell-seeded matrix constructs were collected and fixed in 4% formalin solution. Subsequently, the samples underwent dehydration using ethanol at 4 °C in gradient concentrations (50%, 70%, and 100%) for 30 min each and were air-dried overnight. Cross-sectional images of the bead matrices were then captured and analysed using Image J analysis software.

## Results

### Bead preparation

**Fig. 2:**
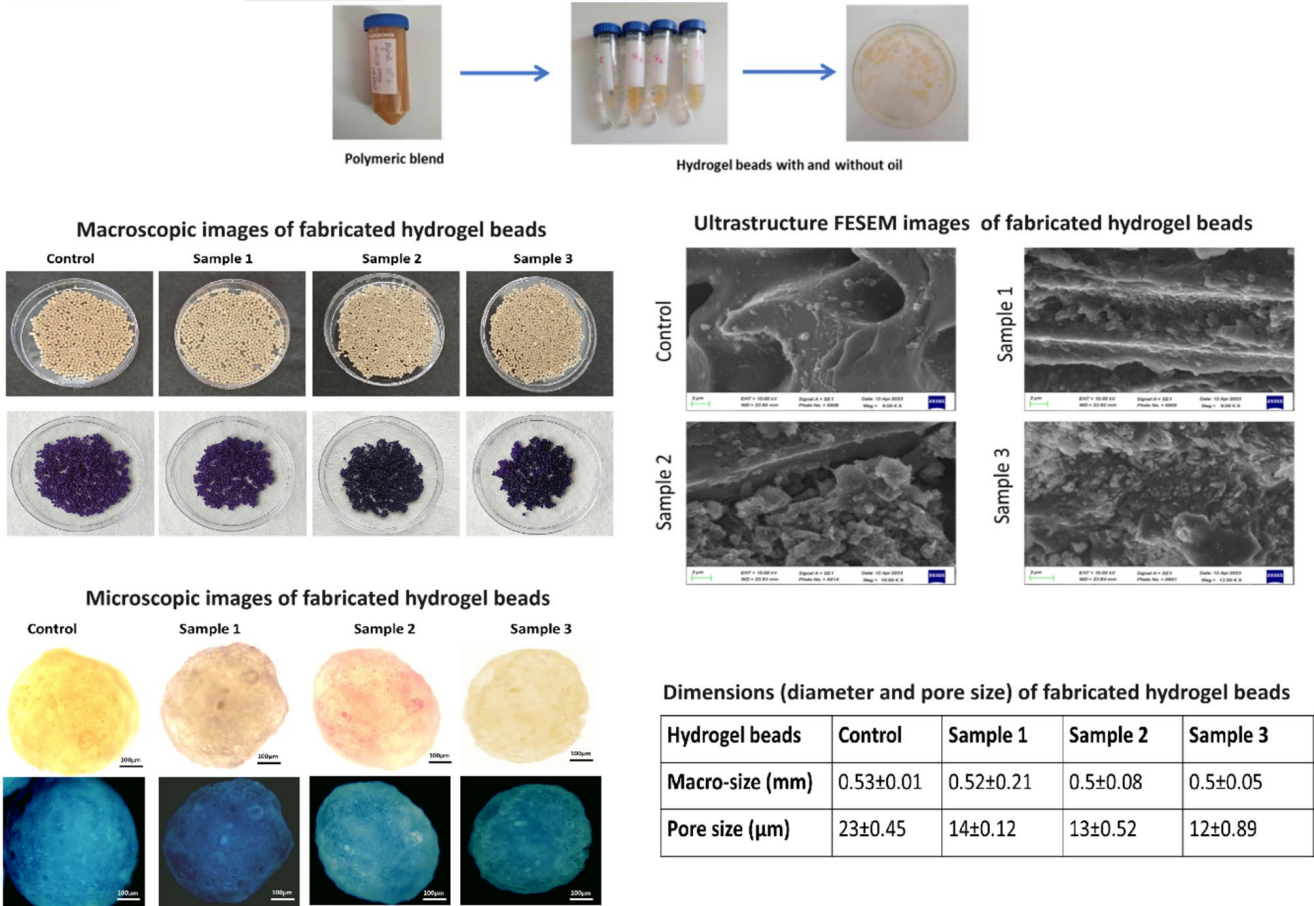
Macroscopic dimension of the fabricated polymeric hydrogel beads: control (0% fish oil), sample 1 (0.1 ml), sample 2 (0.3 ml) and sample 3 (0.5 ml) and their macro and microscopic ultrastructure images of the samples indicating rough porous surface and the deposition the oil content on the surface of the polymeric matrix with reduced pore size.

The morphology and relative size measurements (mean ± SE) of the beads were conducted through simple visualization in microscope (Fig. 2), indicates the average size of the beads are 0.5 mm and no significant variation in their si2ze after entrapment of the oil content was observed. However, FESEM images reveal surface variations in pore size, notably indicating a reduction in pore size after the incorporation of the oil content. This suggests a modification in the surface properties of the beads, with images indicating the deposition of the fish oil's active components on the surface of the polymeric gel bead. Furthermore, gel beads containing higher oil concentrations exhibit maximum deposition, which could prove beneficial for various application. In a study, the cress seed mucilage/chitosan, polymeric hydrogel used to fabricate for oil encapsulation showed the spherical shape particles of 9.4 ± 1.2 µm, with rough surface^21^. The encapsulation of oil in the natural polymeric bead system act as compatible matrix for their delivery other than standard matrix. In this study, the particle size of the coacervate droplets were found to be influenced by the alginate-orange peel extract volume ratio and the variable oil concentration. Generally, higher concentrations of fish oil specific volume ratios led to smaller particle sizes. Precisely, at a higher alginate-orange peel extract lower volume ratio of 1:1 and a fish oil concentration of upto 3% (w/v), the average particle size was measured at 0.5 mm with pore size 12 µm. The Fish oil added to the solution, act as a core material for encapsulation within the coacervate droplets, which alters the composition of the solution and may affect the charge distribution within the system, thereby influencing the conditions favoring complex coacervation.

### FTIR analysis

**Fig. 3:**
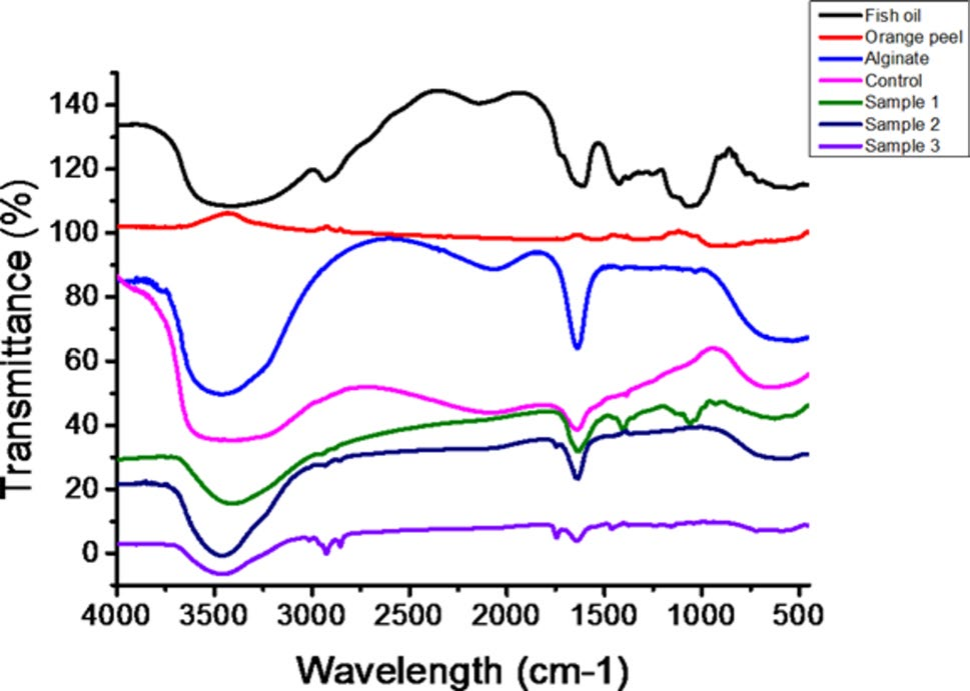
FTIR spectra analysis of pure components and the composite fabricated polymeric beads, ranging from sample control (0% fish oil) to sample 3 (0.5 ml fish oil), confirms the presence of essential peaks and interactions among functional groups.

The investigation focused on the encapsulation of fish oil using alginate-orange peel extract coacervates to ascertain their efficacy in controlling particle size, encapsulation efficiency, and encapsulation load. Results underscored the impact of variations in biopolymer concentration, volume ratio, and core material content on these parameters. FTIR analysis provided valuable insights into the structural features and interactions among the encapsulation components, highlighting modifications and cross-linking between functional groups. FTIR spectra analysis is utilized to elucidate the structural features of components, their compatibility, and interactions, often involving hydrogen bonding (as depicted in Fig. 3). In fish oil, peaks at 3559 cm^−1^, 1762 cm^−1^, and 1108 cm^−1^ suggest stretching vibrations in carboxyl group C=O bonds and C–H double bonds. Similarly, orange peel extract exhibits prominent peaks at 3300 cm^−1^, 1750 cm^−1^, 1267 cm^−1^, and 1001 cm^−1^, indicative of various functional groups. Peaks at 3445 cm^−1^ and 1729 cm^−1^ in the alginate component denote the presence of glucuronic and mannuronic acid units. Modification results in the disappearance and stretching of peaks in control samples, signaling cross-linking between groups. Notably, control samples (sample 1, 2, and 3) show distinct peaks at 1700 cm^−1^, corresponding to C=O stretching in carboxyl groups, and at 3500 cm^−1^. The primary vibrational bands observed in the fish oil spectrum were at 2923 cm^−1^ and 2854 cm^−1^, representing the asymmetric and symmetric stretching of CH2 groups, respectively, characteristic of total polyunsaturated fatty acids (PUFAs)^3^. Additionally, several stretching bands, peaks within the fingerprint region of the fish oil, alginate and orange peel composition were reported in the given spectra^32,36^.

Overall, the study underscores the promising potential of alginate-orange peel extract coacervates as a versatile encapsulation system for bioactive compounds like fish oil, offering applications across functional food, pharmaceuticals, and nutraceuticals. Future research endeavors could delve into optimizing encapsulation parameters and exploring the long-term bioavailability and stability of encapsulated compounds.

### Oil encapsulation efficiency

**Fig. 4:**
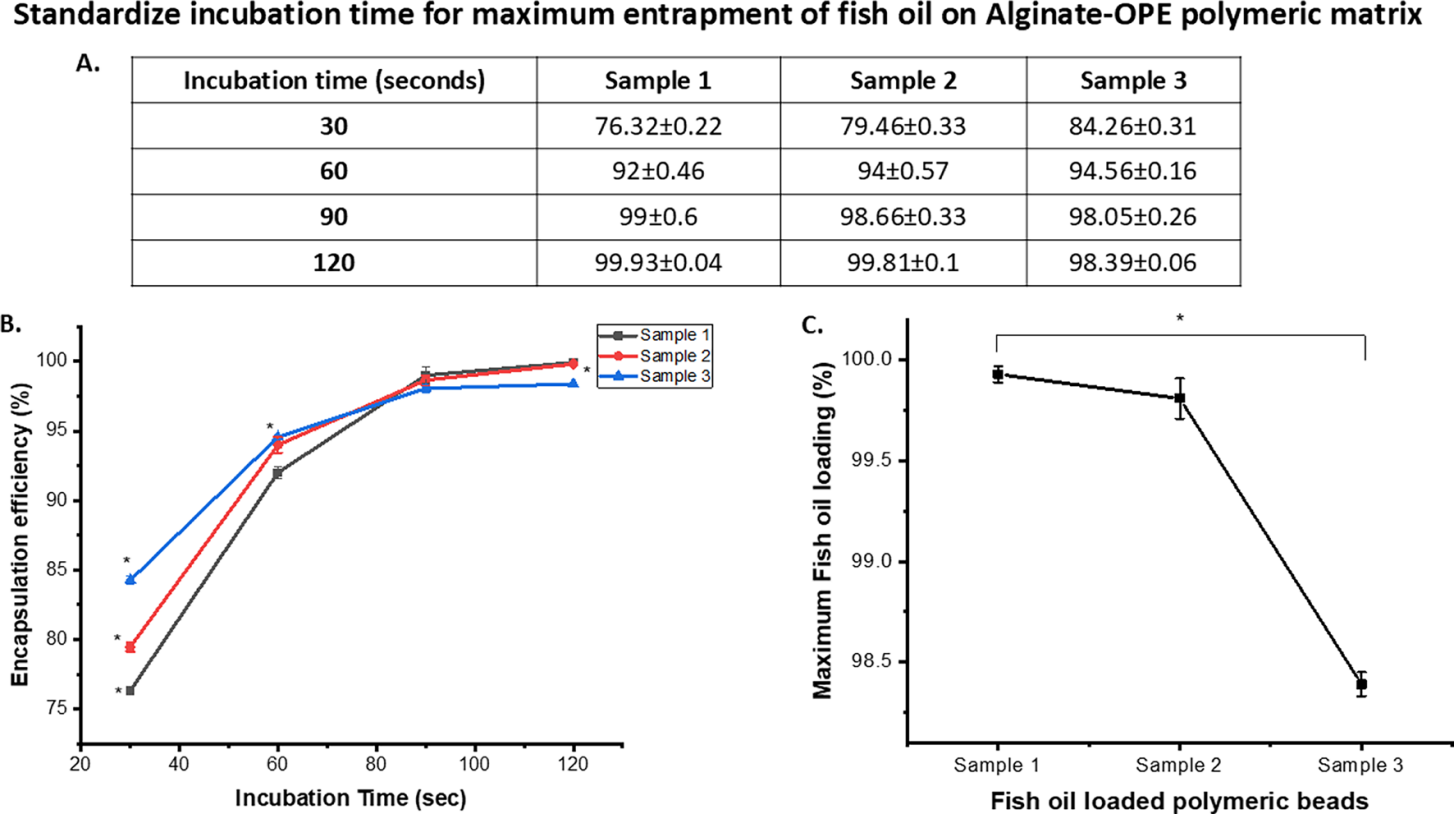
(**A**) Table indicating the incubation time for the maximum entrapment of oil on polymeric beads; (**B**) the release profile with progression of time and (**C**) significant variation (*) in the oil loading efficiency of polymeric gel beads indicates the concentration dependent encapsulation efficiency after 120 s incubation. Here * indicates the significant difference for the *p* value ≤ 0.05.

The ratio of the alginate-OPE polymeric blend had a considerable impact on the encapsulation efficiency of the oil concentration. The constant concentration of alginate-OPE maintained and varying the fish oil concentration content resulted in decreasing effect of efficiency of the encapsulated drug due to the lesser space available to be occupied and encapsulated for the fish oil (Fig. 4). Viscosity is also a crucial parameter to be noted since high viscosity level will lead to the lesser extent of entrapment of the fish oil droplets. The saturation of oil encapsulation obtained after 120 s incubation time with variable sorption rate of oil concentration from 0.1 to 0.5 ml (Fig. 4A,B). According to the result, it can be inferred that Sample 3 with higher fish oil content showed a relatively lower extent of encapsulation (98.39%) while sample 1 and 2 showed an encapsulation efficiency of upto 99% (Fig. 3C). Other than it, the incubation time also effects the encapsulation efficiency, here we found 2 min (120 s) incubation is sufficient for maximum encapsulation of fish oil complete encapsulation. The efficiency of encapsulation depends on the ability of the coacervate phase to encapsulate the core material effectively. Factors such as the composition of the coacervate phase (influenced by biopolymer concentrations and volume ratios) and the properties of the core material (e.g., solubility) can affect encapsulation efficiency^27^. The combined use of alginate and orange peel extract demonstrated synergistic effects in the encapsulation process and robust encapsulation system could enhanced antioxidant protection and improved stability of the encapsulated fish oil. The synergistic interaction potentially resulted in superior encapsulation efficacy and prolonged shelf life of the final product.

### Weight loss, swelling ration and oil release profile

**Fig. 5:**
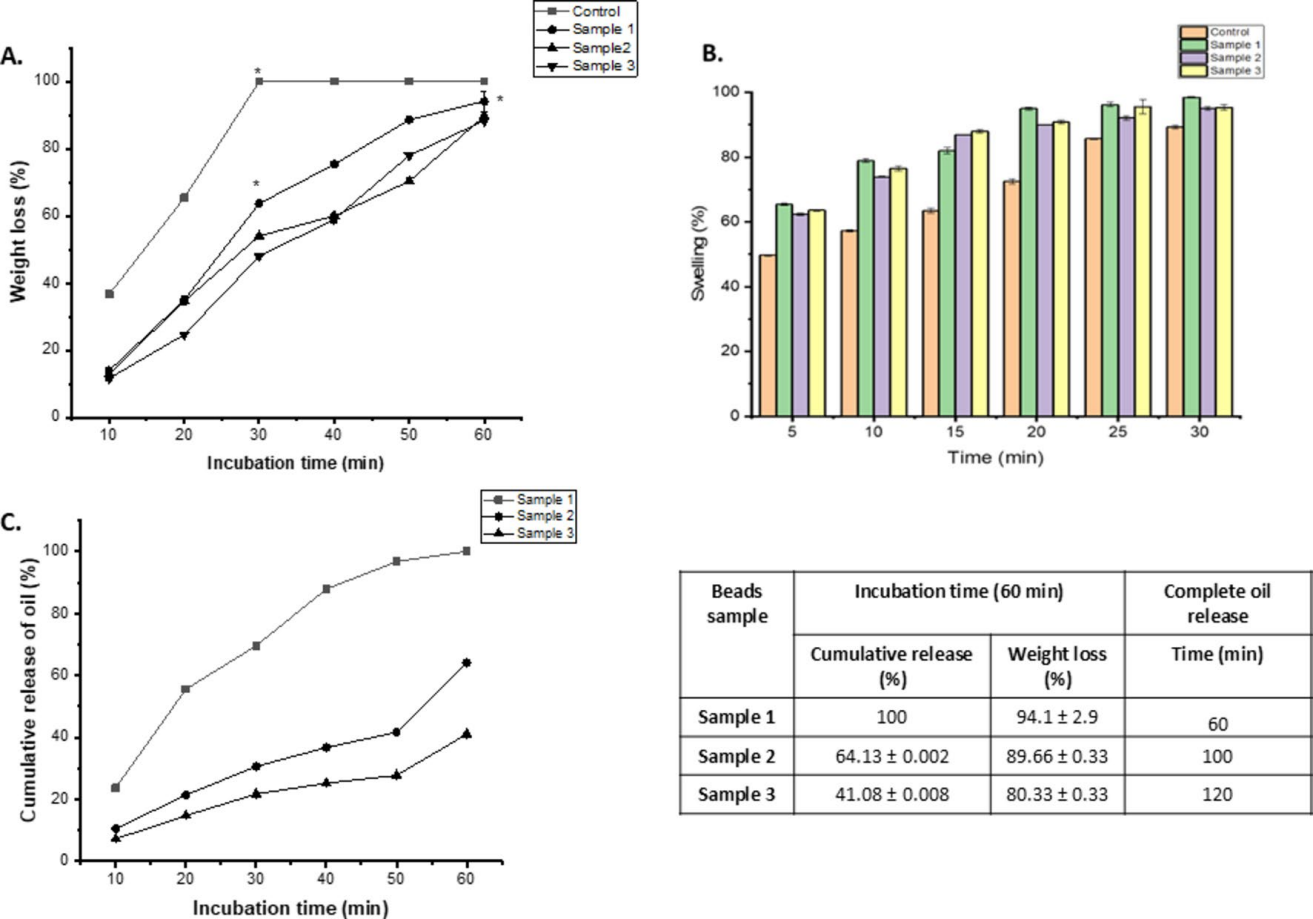
(**A**) In vitro biodegradation; (**B**) swelling profile and (**C**) cumulative release profile of the oil from the fabricated polymeric gel beads: control and oil encapsulated beads in the presence of PBS 1X solution indicates complete swelling along with maximum weight loss within 120 min. Here * indicate the signficant difference among the samples, p value ≤ 0.05.

In vitro biodegradability property was analyzed by ascertaining the level of weight loss occurred in the beads within a duration of 30 min time interval (Fig. 5A). Distinct biodegradation was observed for the differential samples from 30 to 120 min incubation time under constant mechanical agitation. Control sample with no loaded drug showed a degradation of 36% at the first 10 min duration, 65% in the next 10 min and continuing the process till its final complete degradation in 30 min. The samples with loaded drugs showed a slower degradation property due to hydrophobic nature after entrapment of oil. Sample 1 with 0.1 mL of fish oil drug was determined to be degraded at 14% for the first 10 min resulting in 35% degradation in the next duration while giving rise to its complete degradation with 60 min time interval. Although the beads containing 0.3 ml fish oil, sample 2 degradation rate slightly lower than sample 1 and control beads i.e., of 13% to 34% in the next duration of 20 min, and complete degradation within 100 min. Sample 3 showed the least extent of degradation duration of time with its 0.5 mL content of drug pertaining to 11%, 24% and 48% with 10 min gap of time duration, although approximately ≤ 120 min taken for complete degradation. It can be concluded from this result that biodegradation of the beads decreased with the increase loaded oil content, indicates enhanced stability of the beads for prolonged stability and release of the active components in controlled release.

The swelling ratio of each of the samples was determined with the relative analysis of their hydrophilic feature. Immersing of the beads loaded drugs in PBS solution and subsequently recovering them with every five min interval of time is the basis of determining the swelling ratio (Fig. 5B). From the result, it can be concluded that control sample showed 9.7% swelling property at the initial 5 min of duration with a subsequent percentage of 17.3 ± 4.74, 23.4 ± 4.24, 32.6 ± 8.78, 35.6 ± 7.21 and 39.3 ± 5.45 within a gap time interval of 5 min. Sample 1, sample 2 and sample 3 comprising of an increased rate of fish oil content showed a relatively lesser swelling ratio which is attributed to the oil content.

High omega-3 encapsulation efficiency, measured at 92.4 ± 2.3%, was achieved for fibers produced from a blend comprising 10.5% (w/w) polyvinyl alcohol (PVA) and 5% (w/w) emulsion stabilized with whey protein isolate (WPI). This resulted in an impressive oil load capacity of 11.3 ± 0.3%. Examination revealed that the encapsulated oil was distributed in the form of small droplets randomly dispersed within the fibers. Despite these promising encapsulation characteristics, it was observed that the electrospun fibers exhibited elevated levels of hydroperoxides and secondary oxidation products, including 1-penten-3-ol, hexanal, octanal, and nonanal, in comparison to emulsified and unprotected fish oil^30^.

In various studies, biodegradable polymers used in microencapsulation systems highlighting their potential to address concerns over synthetic polymers and emphasizes the advantages such as their mechanical properties and minimal cytotoxicity, while acknowledging the ongoing challenges in manipulating their degradation patterns to avoid toxic byproducts^31^. In brief, the synthesized alginate-orange peel extract are biodegradable polymers are very suitable for the development of new microencapsulation systems with merits of biodegradability and biocompatibility. It can be easily excreted out of body or into nature due to the ability to be eroded in small residues, which is not the case with nondegradable polymers.

The oil release profile was analysed by immersing the sample beads containing varying amounts of fish oil content in a phosphate-buffered saline (PBS) solution, maintaining a pH of 7.4 at room temperature (Fig. 5C). Over a duration of 120 min, the release profile of all samples was studied, revealing a progressive release pattern characterized by rapid initial release followed by a relative decrease in release rate over time.

Specifically, during the first 50 min, a substantial amount of oil was released from all samples. However, the rate of release decreased gradually over the subsequent duration of the experiment. Notably, sample 2 and 3 containing higher amount of fish oil content, exhibited a relatively lower release of the oil, with 30% and 21% of the total content released within the 30 min incubation. In comparison, samples 1 with lower oil content, showed a release percentage of 69% each during the same time frame due to the more hydrophilic nature and swelling ration. Subsequently, the prolonged incubation of the sample under stable conditions results prolonged sustained release of the oil and complete release within 120 min (as shown in Fig. 5).

In the literature, the application of polymeric hydrogels for oil encapsulation is extensively documented, offering a broad spectrum of advantages including controlled release, enhanced stability, and improved bioavailability, rendering them indispensable tools across diverse sectors such as pharmaceuticals, food and nutraceuticals, cosmetics, and various industrial applications^28^. The controlled release properties of polymeric hydrogels enable precise delivery of encapsulated oils, ensuring optimal dosing and prolonged therapeutic effects in pharmaceutical formulations. Furthermore, the enhanced stability provided by hydrogel encapsulation protects oils from degradation due to environmental factors, preserving their integrity and efficacy over extended periods. Additionally, the improved bioavailability facilitated by hydrogels enhances the absorption and utilization of encapsulated oils, thereby maximizing their therapeutic benefits^29^. Such versatile applications highlight the significance of polymeric hydrogels as effective carriers for oil encapsulation, addressing a myriad of challenges and offering innovative solutions in diverse fields.

From these observations, it can be deduced that the release of the oil is influenced by the amount of oil loaded into the beads. Samples with higher oil content demonstrated a relatively higher rate of release, suggesting a direct correlation between drug content and release rate. These findings provide valuable insights into the controlled release behaviour of the encapsulated oil and inform further optimization of encapsulation parameters for desired release kinetics.

### In vitro anti-inflammatory and anti-oxidation analysis

**Fig. 6:**
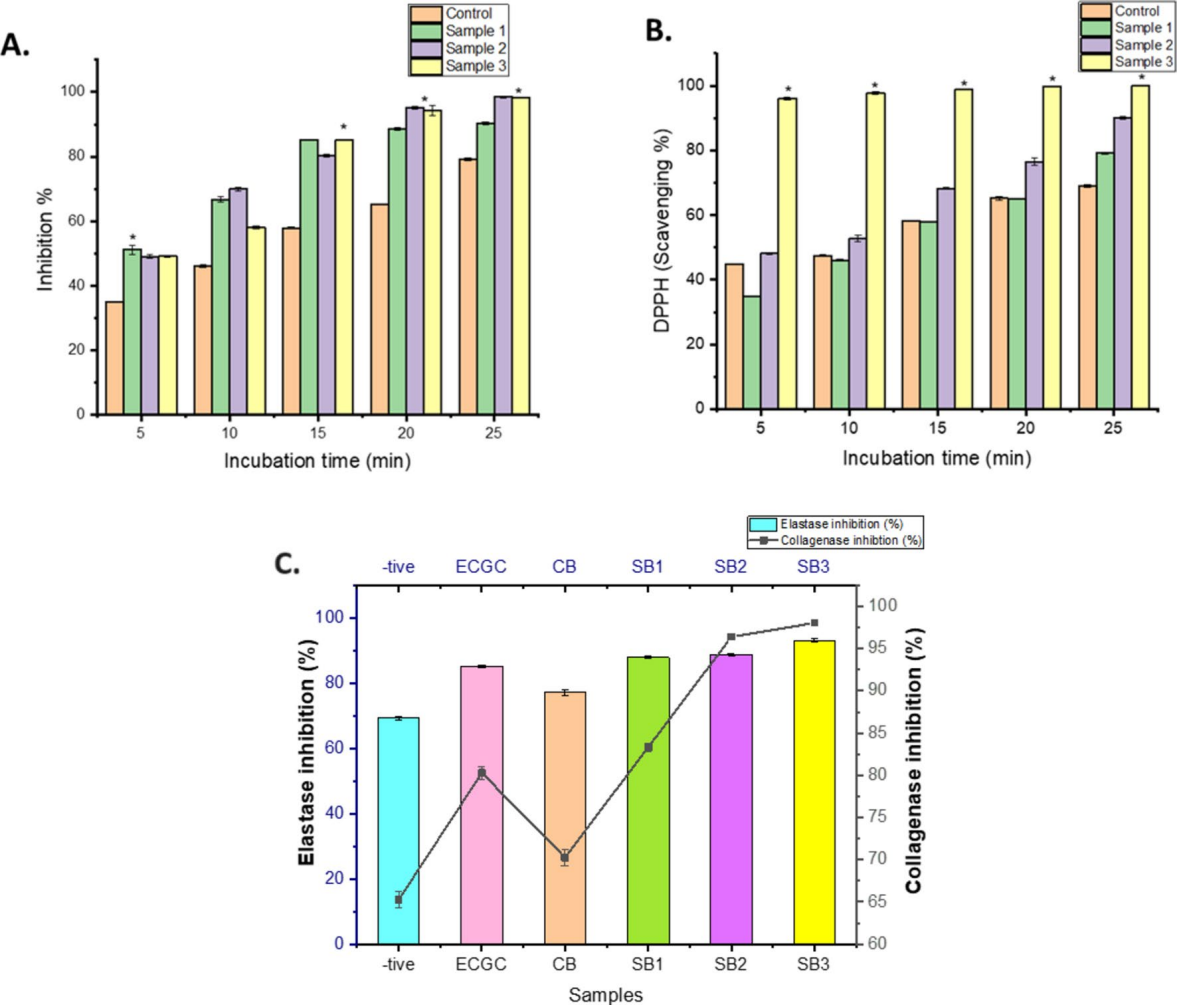
In vitro (**A**) anti-inflammatory and (**B**) anti-oxidative profiling of the gel beads at different time interval with indicates significant difference between the control and oil-gel beads. (**C**) Anti-aging analysis by measuring collagenase and elastase inhibition (%) of the testing sample *(-tive control: Ascorbic acid for collagenase and DMSO for elastase inhibition; ECGC as positive control; CB: control Alginate-OPE beads; SB1, SB2 and SB3 are Alginate-OPE-oil beads containing variable concentration of fish oil i.e., (0.1 *ml*, 0.3 *ml* and 0.5* ml) after 30 min incubation. Here * indicate the signficant difference among the samples, p value ≤ 0.05.

The results from the LOX assay demonstrate that the inclusion of orange peel extract notably enhances the anti-inflammatory response (Fig. 6A). Notably, the control sample, containing citrus extract at a concentration of 2gm/mL without fish oil, exhibited the highest level of protection, reaching 99.5%. In contrast, as the fish oil content increased in subsequent samples, the percentage of protection decreased, indicating a correlation between fish oil presence and diminished membrane protection. These findings underscore the influential role of fish oil in modulating membrane protection and highlight the potential of orange peel extract as a protective agent against hypotonic stress.

The DPPH radical scavenging activity of fish oil encapsulated in hydrogel beads at different concentrations indicates the effectiveness of the encapsulation in preserving the antioxidant properties of the fish oil upto 90% (Fig. 6B). Encapsulation can help protect sensitive compounds like fish oil from degradation and oxidation, thus maintaining their ability to scavenge free radicals effectively. The concentration-dependent response suggests that higher concentrations of fish oil encapsulated in hydrogel beads may result in significant enhanced radical scavenging activity, which can be indicative of the potential utility of such formulations as antioxidants or in other functional applications.

In order to evaluate the enzyme inhibition activity, alginate-OPE polymeric beads with or without fish oil were found to have anti-aging properties. While the samples' absorbance was evaluated after 30 min of incubation, it revealed strong anti-collagenase and anti-elastase action. When the concentration of oil in the bead samples was raised, the polymeric beads' high collagenase and elastase inhibitory activity was seen, compared to the negative control samples (Fig. 6C). As a positive control, epigallocatechin gallate (EGCG) demonstrated about equivalent levels of enzyme inhibition to beads containing 0.1 ml oil 80.27 ± 0.73% anti-collagenase and 85.12% ± 0.51% anti-elastase activity. On the other hand, 70.27 ± 1.0% and 77.17 ± 0.92% anti-collagenase and anti-elastase activity respectively, for the control beads. However, the oil-encapsulated beads in Sample 1 are 83.33 ± 0.48 and 87.94 ± 0.25, Sample 2 are 96.30 ± 0.27 and 88.71 ± 0.25, and Sample 3 are 98.33 ± 0.27 and 93.07 ± 0.44 have an anti-elastase and anti-collagenase% activity. Therefore, the results suggested that the combination of fish oil and Alginate-OPE alginate polymeric beads may have synergistic effects that could potentially have an anti-aging impact. In order to increase the potency of the beads as an active ingredient in anti-aging products, their inhibitory concentration was significantly higher than that of the control samples. This proved to be effective, as the results demonstrated even higher levels of anti-elastase and anti-collagenase activity.

In several studies, to enhance the functional properties of fish oil and its derivatives by incorporating them into various formulations with added benefits such as increased antioxidant activity, improved oxidative stability, and antibacterial properties^32,33^. These findings could have implications for developing value-added food products with improved nutritional profiles and health benefits**.**

### In vitro cell culture

**Fig. 7:**
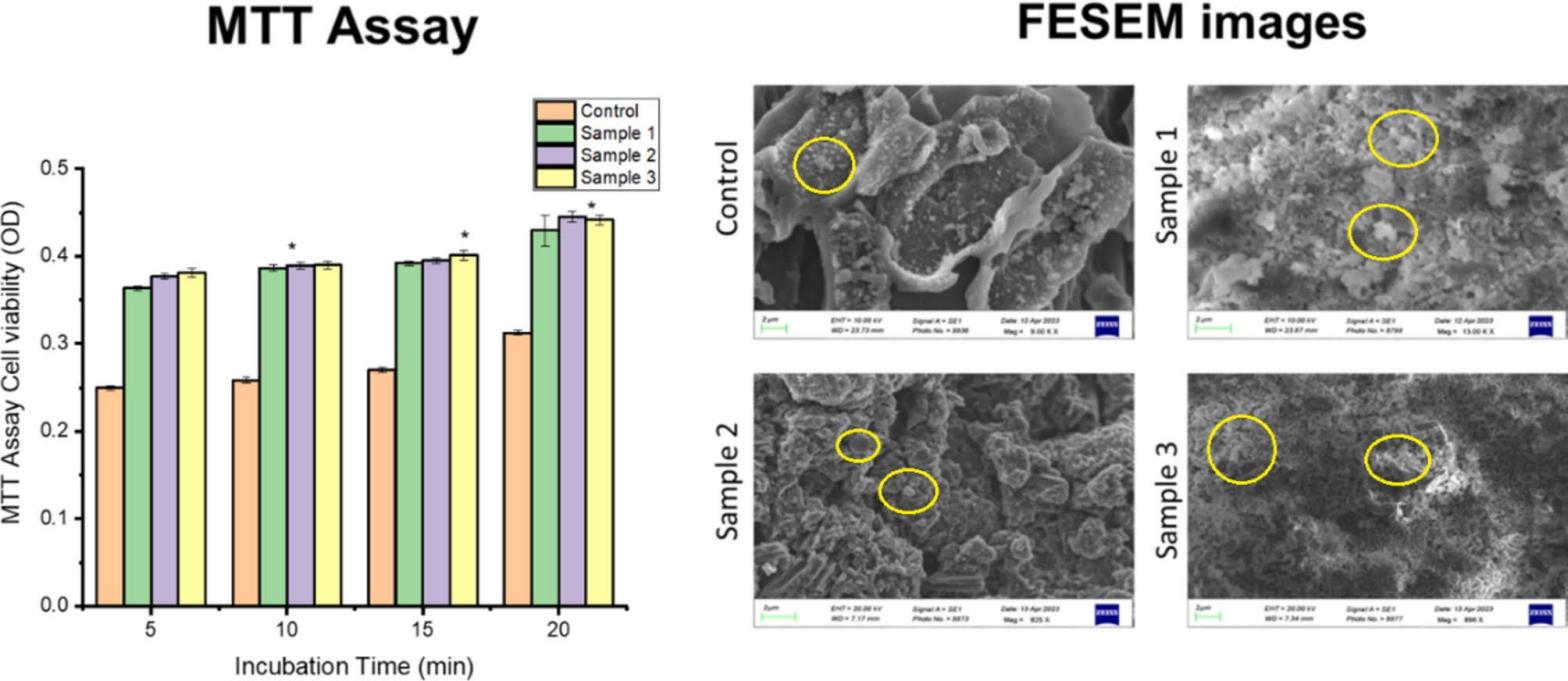
In vitro assessment of the cell seeded gel beads by MTT assay analysis and the FESEM images analysis indicates positive impact on cell viability, differentiation and growth, * indicates the significant difference for the p value ≤ 0.05.. Here proliferated cells indicated by yellow circle labelling.

MTT assay and FESEM analysis play crucial roles in various research areas, including drug discovery, biomaterials development, tissue engineering, and nanotechnology, providing valuable insights into cellular responses and material characteristics at the micro- and nanoscale levels, respectively^34^. The in vitro assessment of gel bead biocompatibility and their impact on cell viability was conducted using MTT assay analysis and morphology analysis of cell-seeded beads. The results revealed a positive effect on cell growth, viability, indicates gel beads exhibited high metabolic activity and excellent biocompatibility (Fig. 7). The high absorbance value indicates higher cell proliferation and growth over the matrix, with the progression of time the absorbance value increased showed constant cell division. The oil entrapped gel beads have significant effect on cell growth and faster cell proliferation with progression time and concentration. Sample 2 and 3 beads containing 3 µl and 5 µl oil /ml of polymeric blend indicates similar growth rate within 20 min incubation. Furthermore, FESEM microscopic examination images of the cell-seeded beads for the morphology analysis demonstrated the favorable cell growth and attachment on the surface of the cell-seeded beads confirming their compatibility and promoting cell proliferation. The neo-ECM matrix's deposition on the gel beads' surface demonstrates favorable cell morphology, attachment, and differentiation, emphasizing their capacity to promote cellular interactions and growth along with increased cell proliferation. This validates the fabricated beads' cytocompatibility and regenerative potential.

Among all the bead samples, the cells seeded over the fish oil encapsulated matrices exhibit higher viability compared to cells treated with non-encapsulated fish oil, indicates the protective effect of the encapsulation on cellular health. Thus, we can conclude that the the effects of fish oil encapsulated matrices on cellular behavior vary depending on the concentration or formulation used and at concentration of 0.3 ml sample 2 as higher doses often eliciting more pronounced responses. This demonstrates the importance of dosage optimization for achieving desired biological outcomes. Overall, these findings highlight the potential of gel beads for biomedical applications where biocompatibility and support for cell growth are essential**.** These findings collectively suggest the potential of cell-seeded gel beads for tissue engineering and regenerative medicine applications, where the promotion of cell viability and differentiation is crucial for successful outcomes.

The research findings cover diverse applications of polymers across different fields of sciences and technology such as Omeg@Silica microparticles show promise in lung cancer therapy, outperforming fish oil in inducing favorable effects on cancer cells^35^. Multi-layered microcapsules with trace fish oil enhance probiotic resilience, while challenges remain in fabricating ultra-low concentration sodium alginate/pectin hydrogels for cell growth (Huang et al., 2021). In the food industry, alginate/LPI microcapsules with Cod liver oil improve meat products by enhancing stability and sensory qualities^36^. Hydrogels with reduced polymer content have been shown to enhance 3D cell growth. However, creating ultra-low concentration gelatin hydrogels presents challenges, particularly in achieving rapid gelation and ensuring stability^37^. These findings underscore interdisciplinary innovation's importance in addressing challenges and advancing various sectors for societal benefit.

## Conclusion

In conclusion, the synthesis of novel polymeric alginate-orange peel extract alginate hydrogel and its application for encapsulating fish oil demonstrate promising potential for biomedical and pharmaceutical applications. The encapsulation process yielded high encapsulation efficiency of up to 98% within a short incubation period, with controlled release capabilities observed. Additionally, Fourier-transform infrared spectroscopy confirmed successful encapsulation and interaction between constituents, highlighting the robustness of the hydrogel formulation.

Considering the advanced research and technology to assist the efficient and precise therapeutic effect of these natural anti-inflammatory products, encapsulation serves as a delivery technology. This type of drug delivery system is a good candidate for susceptible substances and their entrapment. The hydrogel beads showed no significant decrease in size and a notable reduction in pore size, indicating favorable physical characteristics. Moreover, in vitro degradation studies demonstrated the hydrogel's biodegradability within 30 min, suggesting its potential for environmentally friendly applications. Furthermore, the hydrogel exhibited anti-inflammatory and anti-oxidative activities, contributing to enhanced biocompatibility.

The use of natural anti-inflammatory products, such as those encapsulated within the hydrogel beads, offers a promising alternative to synthetic drugs like NSAIDs, mitigating potential side effects while providing comparable therapeutic effects. Encapsulation technology enhances the therapeutic efficacy of these natural compounds, offering synergistic effects for improved treatment outcomes.

Overall, this comprehensive approach involving in vitro evaluations underscores the potential of the synthesized hydrogel formulation as a versatile drug delivery system. Further research and optimization of this system may lead to its broader application in drug delivery and tissue engineering, offering promising therapeutic options for various inflammatory conditions and oxidative stress-related disorders.

## Data Availability

The datasets used and/or analysed during the current study available from the corresponding author on reasonable request.
